# Inadequate Exercise as a Risk Factor for Sepsis Mortality

**DOI:** 10.1371/journal.pone.0079344

**Published:** 2013-12-04

**Authors:** Paul T. Williams

**Affiliations:** Donner Laboratory, Life Sciences Division, Ernest Orlando Lawrence Berkeley National Laboratory, Berkeley, California, United States of America; Boston University, United States of America

## Abstract

**Objective:**

Test whether inadequate exercise is related to sepsis mortality.

**Research Design and Methods:**

Mortality surveillance of an epidemiological cohort of 155,484 National Walkers' and Runners' Health Study participants residing in the United States. Deaths were monitored for an average of 11.6-years using the National Death index through December 31, 2008. Cox proportional hazard analyses were used to compare sepsis mortality (ICD-10 A40-41) to inadequate exercise (<1.07 METh/d run or walked) as measured on their baseline questionnaires. Deaths occurring within one year of the baseline survey were excluded.

**Results:**

Sepsis was the underlying cause in 54 deaths (sepsis_underlying_) and a contributing cause in 184 deaths (sepsis_contributing_), or 238 total sepsis-related deaths (sepsis_total_). Inadequate exercise was associated with 2.24-fold increased risk for sepsis_underlying_ (95%CI: 1.21 to 4.07-fold, P = 0.01), 2.11-fold increased risk for sepsis_contributing_ (95%CI: 1.51- to 2.92-fold, P<10^−4^), and 2.13-fold increased risk for sepsis_total_ (95%CI: 1.59- to 2.84-fold, P<10^−6^) when adjusted for age, sex, race, and cohort. The risk increase did not differ significantly between runners and walkers, by sex, or by age. Sepsis_total_ risk was greater in diabetics (P = 10^−5^), cancer survivors (P = 0.0001), and heart attack survivors (P = 0.003) and increased with waist circumference (P = 0.0004). The sepsis_total_ risk associated with inadequate exercise persisted when further adjusted for diabetes, prior cancer, prior heart attack and waist circumference, and when excluding deaths with cancer, or cardiovascular, respiratory, or genitourinary disease as the underlying cause. Inadequate exercise also increased sepsis_total_ risk in 2163 baseline diabetics (4.78-fold, 95%CI: 2.1- to 13.8-fold, P = 0.0001) when adjusted, which was significantly greater (P = 0.03) than the adjusted risk increase in non-diabetics (1.80-fold, 95%CI: 1.30- to 2.46-fold, P = 0.0006).

**Conclusion:**

Inadequate exercise is a risk factor for sepsis mortality, particular in diabetics.

## Introduction

Sepsis is a deleterious host reaction that occurs when a systemic inflammatory response becomes dysregulated and immunosuppression follows, which can lead to hypotension, organ dysfunction, and death [1]. Although usually caused by staphylococci or streptococci infections, it can also be caused by other bacteria, fungi, viruses, or parasites [1]. Clinically, sepsis diagnosis requires a suspected or proven underlying infection with two or more findings of systemic inflammatory response syndrome (*e.g.*, fever, tachycardia, tachypnea, leukocytosis) that cannot be attributed to other causes [2]. Sepsis accounts for approximately 9% of all deaths in the United States, and has an overall hospital mortality of about 30% [3]. It substantially increases long-term mortality [4], [5] and cognitive impairment [6], and decreases functionality [6] and quality of life [5]. The incidence of sepsis is projected to increase 1.5% annually and to affect 1.1 million in 2020 [3]. Some reports estimate the annual increase in the incidence of sepsis to be between 5% and 9% in the United States [7].

Currently recognized risk factors for sepsis include older age, male sex, being of African descent or other non-Caucasian race, and co-morbidities [3], [7]. Sepsis mortality is greater in the elderly and HIV-infected, and is much lower in previously healthy adults [3], [7]. Delayed or inappropriate treatment is also a major risk factor for sepsis mortality, with increased mortality for each hour delay in the administration of effective antibiotics [8]. Although physical inactivity and inadequate exercise have not traditionally been considered risk factors for sepsis, those who exercise are generally in better health, and are at lower risks for some co-morbidities associated with sepsis [9]–[12]. Physical activity is also associated with improved immune response [13]–[15], and animal models suggest prior exercise training improves the inflammatory response, and reduces organ dysfunction and mortality following sepsis induction [16]–[20], suggesting that physical activity and exercise may also decrease sepsis risk directly.

Analyses were therefore performed to assess the association of running and walking with sepsis mortality prospectively in 155,484 participants of the National Walkers' and Runners' Health Studies [10]–[12], [21]–[23] which are the largest epidemiological cohorts specifically designed to study exercise and health.

## Materials and Methods

The National Walkers' and Runners' Health Studies have been described in detail [13]–[16]. Walkers were recruited between 1999 and 2001, while runners were recruited in two waves, between 1991 and 1993 (phase I) and between 1998 and 2001 (phase II), through solicitation of subscribers of activity-targeted publications and participants at footrace events [10]–[12], [21]–[23]. The study protocol was approved by the University of California Berkeley committee for the protection of human subjects, and all subjects provided a signed statement of informed consent.

Participants completed baseline questionnaires on exercise, height, current and past body mass, body circumferences, diet, current and past cigarette use, and history of disease. Diabetes was defined as reported diabetes medication use on the baseline survey. The runners reported the usual miles run per week, and the walkers reported usual miles walked per week and usual pace (minutes per mile). These were used to estimate energy expenditure in terms of metabolic equivalents (MET), where one MET is the energy expended sitting at rest (3.5 ml O_2_•kg^−1^•min^−1^, 1 MET-hour ∼1 km run) [24]. In walkers, METh/d walked was calculated by converting reported distances into durations (i.e., distance/mph), which were then multiplied by the MET value for the reported pace [21], [23]. In runners, METh/d run was calculated as km run*1.02 METh/km [22], [23]. Previously, we have reported strong correlations between repeated questionnaires for self-reported running distance (r = 0.89) [25].

Education was obtained by requesting that the participant provide “years of education (examples: HS = 12; BS or BA = 16; MS or MA = 18; PhD or MD = 20).” Height and body mass were determined by asking the participant, “What is your current height (in inches, without shoes)?” and, “What is your current weight (pre-pregnancy weight if pregnant)?” Body mass index (BMI) was calculated as body mass in kilograms divided by the square of height in meters. Self-reported waist circumference was obtained in response to the request, “Please provide, to the best of your ability, your body circumference in inches: waist___, hip___, and chest___,” without further instruction. Elsewhere, we have reported the strong correlations between self-reported and clinically measured heights (r = 0.96) and body masses (r = 0.96) [25]. Self-reported waist circumferences were somewhat less precise, as indicated by their correlation with reported circumferences on a second questionnaire (r = 0.84) and with their clinical measurements (r = 0.68) [25]. Other data collected in our clinic suggest little difference in the mean waist circumference from survey questionnaires and their clinic measurements.

Intakes of meat and fruit were based on the questions “During an average week, how many servings of beef, lamb, or pork do you eat”, and “…pieces of fruit do you eat”. Alcohol intake was estimated from the corresponding questions for 4-oz. (112 ml) glasses of wine, 12-oz. (336 ml) bottles of beer, and mixed drinks and liqueurs. Alcohol was computed as 10.8 g per 4-oz glass of wine, 13.2 g per 12 oz. bottle of beer, and 15.1 g per mixed drink [26].

Underlying and contributing (entity axis) causes of death were obtained from the National Death Index mortality surveillance through December 31, 2008 [27]. Possible matches between cohort members and the National Death Index database needed to agree on at least one of the following conditions: 1) Social Security number; 2) exact month and ±1 year of birth, first and last name; 3) exact month and ±1 year of birth, first and middle initials, last name; 4) exact month and day of birth, first and last name; 5) exact month and day of birth, first and middle initials, last name; or 6) exact month and year of birth, first name, and last name with the father's surname on the National Death Index record. Agreement on last names was based on exact spelling or common misspellings. Multiple records were submitted for each participant to cover potential name variations (e.g., nicknames, prior married names). The National Death Index assigned probability scores to potential matches, and the high probability score matches were further reviewed by survey staff for acceptance while blinded to exercise level and other variables that could influence mortality. The National Death Index has shown to identify over 90% of decedents and discrepancy from nosology assigned causes of death in 4% of cases [28].

### Statistics

Cox proportional hazard analyses (STATA version 11.1, StataCorp, College Station, TX) were used to test whether sepsis deaths (International Classification of Disease version 9 code 191, and version 10 code A40 and A41 [29]) were significantly related to METh/d run or walked and other risk factors when adjusted for sex, baseline age (age and age^2^), education, self-identified non-white race, and cohort effects (NRHS-I, NRHS-II, NWHS). Although tests for associations between MET-hours/d of exercise and cause-specific mortality were the primary hypotheses, sepsis was not identified a priori as one of the disease endpoints of interest. Results are presented as hazard ratios (HR), their fold increases in risk, and their percent reductions in the risk (calculated as 100*(HR-1)) for four categories of walking or running energy expenditure: 1) falling short of the current physical activity recommendations for health (<450 MET minutes per week = 1.07 METh/d), 2) meeting the recommendations (450 to 750 MET minutes per week = 1.07 to 1.8 METh/d), 3) exceeding the recommendations by 1- to 2-fold (1.8 to 3.6 METh/d), and 4) exceeding the recommendations by ≥2-fold (≥3.6 METh/d) [24]. Deaths occurring within one year of the baseline survey were excluded. Contingency tables and logistic regression analyses were also used to test whether METh/d run or walked affected the proportion of all death that were sepsis-related, in order to determine whether the increased or decreased risk was merely a reflection of overall shifts in total mortality. The data used in these analyses require human use approval.

## Results

Table 1 presents the baseline characteristics for the 54 subjects who died with sepsis assigned as the underlying cause of death (sepsis_underlying_), 184 subjects who died of some other underlying cause but with sepsis included as contributing (sepsis_contributing_), and the remaining subjects who survived (N = 150,508) or whose deaths were unrelated to sepsis (N = 4738). Thus, 238 deaths included sepsis as either an underlying or contributing cause (sepsis_total_ = sepsis_underlying_+sepsis_contributing_). The 54 sepsis_underlying_ deaths included fifty unspecified sepsis, one group D streptococcus, one Streptococcus pneumoniae, one Staphylococcus aureus, and one unspecified staphylococcus. The 184 sepsis_contributing_ deaths included 166 unspecified sepsis, one group D streptococcus, one Staphylococcus aureus, six unspecified staphylococcus, seven other Gram-negative organisms, and three other sepsis. The underlying causes for the 184 sepsis_contributing_ deaths were: other infections (5), neoplasms (61), malnutrition (2), amyloidosis (1), acidosis (1), mental and behavioral disorders (3), external causes and accidents (4), and diseases of the nervous system (4), circulatory system (27), respiratory system (28), digestive system (25), musculoskeletal system and connective tissue (6), and genitourinary system (12).

**Table 1:** Sample baseline characteristics (percent or mean±SD).

	Sepsis Underlying cause	Sepsis Contributing cause	Other mortality or alive
Sample (N)	54	184	155,246
Mortality surveillance (years)	11.56±3.25	11.10±3.18	11.58±3.15
Runners (%)	44.44%	42.93%	73.03%
Female (%)	38.89	39.13	52.39
Non-white (%)	3.70	14.67	9.62
Age (years)	69.36±11.30	63.91±14.28	44.78±12.78
Education (years)	14.90±2.76	15.17±2.86	15.94±2.60
Years run or walked	17.69±17.85	16.21±13.43	10.77±8.53
BMI (kg/m^2^)	24.43±5.07	25.78±4.78	23.91±4.06
Waist circumference (cm)	82.93±9.61	88.52±13.78	79.43±11.04
Diabetic (%)	11.11	11.41	1.37
Heart attack survivor (%)	20.38	10.33	1.51
Cancer survivor (%)	3.70	19.02	3.45
Smoker (%)	1.85	4.35	2.86
Alcohol (g/d)	10.64±17.25	9.21±17.03	8.76±13.77
Meat (serving/d)	0.34±0.32	0.40±0.44	0.36±0.40
Fruit (pieces/d)	1.60±0.93	1.55±1.28	1.52±1.22

### Dose-response

**Figure 1:**
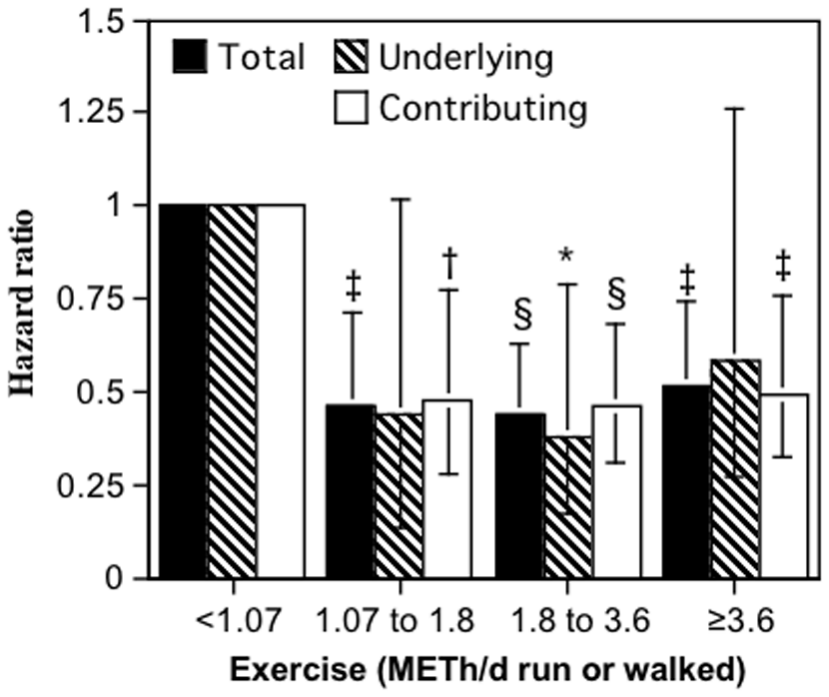
Relative risk of sepsis mortality by METh/d run or walked in 155,484 subjects during an average of 11.6-year mortality surveillance. There was 13.4% of the sample that was inadequately active (<1.07 MET-hours/d), 8.8% that met the exercise recommendations (1.07 to 1.8 MET-hours/d), 29.3% that exceeded the recommendations by 1 to 2-fold, (1.8 to 3.6 MET-hours/d), and 48.5% that exceeded the recommendations by ≥2-fold (≥3.6 MET-hours/d). Brackets designate 95% confidence intervals. Relative risks (i.e., the hazard ratios) were calculated from Cox proportional hazard analyses adjusted for sex, age, race, and cohort effects. Significant risk reductions relative to the inadequate exercise group were coded: * P≤0.05; † P≤0.01, ‡ P≤0.001, and § P≤0.0001.

Figure 1 displays the survival analyses for sepsis risk vs. METh/d run or walked, adjusted for age, sex, and cohort. The results show that the reduction in risk to be similar for sepsis_underlying_ and sepsis_contributing_. Total sepsis mortality (underlying and contributing combined) was 54.0% lower for 1.07 to 1.8 METh/d (95%CI: 28.8% to 71.4% reduction, P = 0.0004), 55.8% lower for 1.8 to 3.6 METh/d (95%CI: 37.5% to 69.0% reduction, P<10^−5^), and 48.6% lower for ≥3.6 METh/d relative to <1.07 METh/d (95%CI: 25.6% to 64.6% reduction, P = 0.0004).

### Inadequate exercise

Figure 1 shows little if any additional risk reduction above 1.07 METh/d, suggesting that the risk for sepsis mortality can be more simply expressed as the risk associated with exercising below the guideline level of 1.07 METh/d (i.e., inadequate exercise). Therefore, compared to achieving or exceeding the guideline level, inadequate exercise was associated with a 2.24-fold greater risk for sepsis_underlying_, 2.11-fold for sepsis_contributing_, and 2.13-fold increase for sepsis_total_ mortality (Table 2). The greater sepsis_total_ mortality for <1.07 vs. ≥1.07 METh/d did not differ significantly between runners and walkers (P = 0.59), males and females (P = 0.37), or by age (P = 0.09 for interaction). The number of years run or walked >19 kilometers per week showed no relationship to sepsis_underlying_ (P = 0.84), sepsis_contributing_ (P = 0.50), or sepsis_total_ mortality (P = 0.51). Average number of marathons run per year was also unrelated to sepsis_underlying_ (P = 0.46), sepsis_contributing_ (P = 0.50), or sepsis_total_ (P = 0.90) in the 113,472 runners.

**Table 2:** Hazard ratios from Cox proportional hazard analyses (95% confidence interval) of sepsis mortality for inadequate exercise vs. achieving or exceeding the exercise recommendations, i.e., <1.07 MET-hours/d vs. ≥1.07 MET-hours/day.

	sepsis_total_	sepsis_underlying_	sepsis_contributing_
All subjects, standard covariates*:			
only	2.13	2.24	2.11
	(1.59, 2.84)	(1.21, 4.07)	(1.51, 2.92)
	P<10^−5^	P = 0.01	P<10^−4^
plus expanded covariates†	1.98	2.33	1.89
	(1.45, 2.69)	(1.18, 4.88)	(1.33, 2.68)
	P<0.0001	P = 0.01	P = 0.0005
plus waist circumference	1.87	2.15	1.81
	(1.32, 2.63)	(0.99, 4.44)	(1.22, 2.66)
	P = 0.005	P = 0.05	P = 0.004
Standard covariates*, excluding deaths due to:			
Cancers‡	2.00	2.24	1.91
	(1.43, 2.77)	(1.20, 4.08)	(1.28, 2.82)
	P = 0.0001	P = 0.01	P = 0.002
cardiovascular diseases‡	2.06	2.27	1.99
	(1.51, 2.80)	(1.22, 4.12)	(1.39, 2.84)
	P = 0.0001	P = 0.01	P = 0.0003
respiratory diseases‡	2.19	2.24	2.19
	(1.61, 2.98)	(1.21, 4.08)	(1.52, 3.12)
	P = 10^−5^	P = 0.01	P<0.0001
genitourinary diseases‡	2.05	2.24	2.00
	(1.51, 2.75)	(1.21, 4.08)	(1.41, 2.80)
	P = 10^−5^	P = 0.01	P = 0.0001
Subjects ≥60 years, standard covariates*	1.79	1.98	1.72
	(1.26, 2.51)	(1.01, 3.79)	(1.14, 2.55)
	P = 0.001	P = 0.05	P = 0.01

* age (age, age^2^), sex, race, exercise (runner vs. walker), and cohort adjusted.

† Expanded covariates include education, diabetes, prior heart attack, prior cancer, and BMI in addition to the standard covariates.

‡ underlying cause.

The greater sepsis mortality was not simply a reflection of the greater total mortality associated with inadequate exercise, *i.e.*, sepsis represented a greater proportion of total deaths for the <1.07 vs. ≥1.07 METh/d group. The proportions of sepsis_total_ deaths in those reporting inadequate (6.86% of the 1239 total deaths) and adequate exercise (4.09% of the 3737 total deaths) were significantly different (2×2 Chi square: P<0.0001). Logistic regression analyses further confirmed the greater involvement of sepsis in deaths occurring in the inadequate vis-à-vis adequate exercise group (P = 0.003) when adjusted for age, sex, race, and cohort.

### Race, education, BMI, diabetes, prior heart attack, and prior cancer

When adjusted for age, sex, cohort, and inadequate exercise, the risk for sepsis_total_ mortality was 1.70-fold greater for non-whites (95%CI: 1.12- to 2.47-fold greater, P = 0.01). When adjusted for race in addition to age, sex, cohort, and inadequate exercise, the risk for sepsis_total_ mortality was 1.93-fold greater for heart attack survivors (95%CI: 1.27- to 2.86-fold greater, P = 0.003), 2.28-fold greater for cancer survivors (95%CI: 1.57- to 2.79-fold greater, P = 0.0001) and 3.01–fold greater for diabetes medication users (95%CI: 1.94- to 4.50-fold greater, P = 10^−5^). Adjusted sepsis_total_ mortality was also 3.32% greater per kg/m^2^ increase in BMI (95%CI: 0.00% to 6.29%, P = 0.05), and 5.9% lower per year of education (95%CI: 1.3% to 10.2% decrease, P = 0.01). Adjustment for race, education, diabetes, prior heart attack, prior cancer, and BMI in addition to age, sex and cohort did not eliminate the increased risk due to inadequate exercise on sepsis mortality (Table 2).

### Abdominal obesity

There were 134,762 subjects (101,351 runners, 33,411 walkers) who reported their waist circumferences (86.7% of the sample). Greater waist circumference increased the risk for sepsis_total_ (2.59% per cm, 95%CI: 1.18% to 3.95%, P = 0.0004, 185 deaths) when adjusted for age, sex, race, cohort, and inadequate exercise, which remained significant when further adjusted for BMI (2.90% per cm, 95%CI: 0.85% to 4.90%, P = 0.006), whereas BMI was unrelated to sepsis_total_ when adjusted for waist circumference (P = 0.66). Those who were abdominally obese (waist circumference >102 cm in men and >88 cm in women) were at 1.74-fold greater risk for sepsis_total_ mortality (95%CI: 1.03- to 2.87-fold, P = 0.04). Inadequate exercise remained a significant predictor of mortality when adjusted for waist circumference in addition to the other covariates (Table 2).

### Co-morbidity

The increased risk of sepsis_total_ from inadequate exercise could not be attributed to its associations with other co-morbidities. Specifically, the risks scarcely changed and significance was retained when deaths due to cancer, cardiovascular disease, respiratory disease, or genitourinary diseases as the underlying cause were excluded (Table 2). In fact, inadequate exercise was associated with significantly greater risk of sepsis being listed as a contributing cause in the 1847 cancer deaths (2.81-fold greater risk, 95%CI: 1.51- to 5.13-fold, P = 0.002, where 56 cancer deaths included sepsis as a contributing cause) and the 1453 cardiovascular disease deaths (3.40-fold greater risk, 95%CI: 1.40- to 8.22-fold, P = 0.007, where 27 cardiovascular disease deaths included sepsis as a contributing cause).

### Diabetics

**Figure 2:**
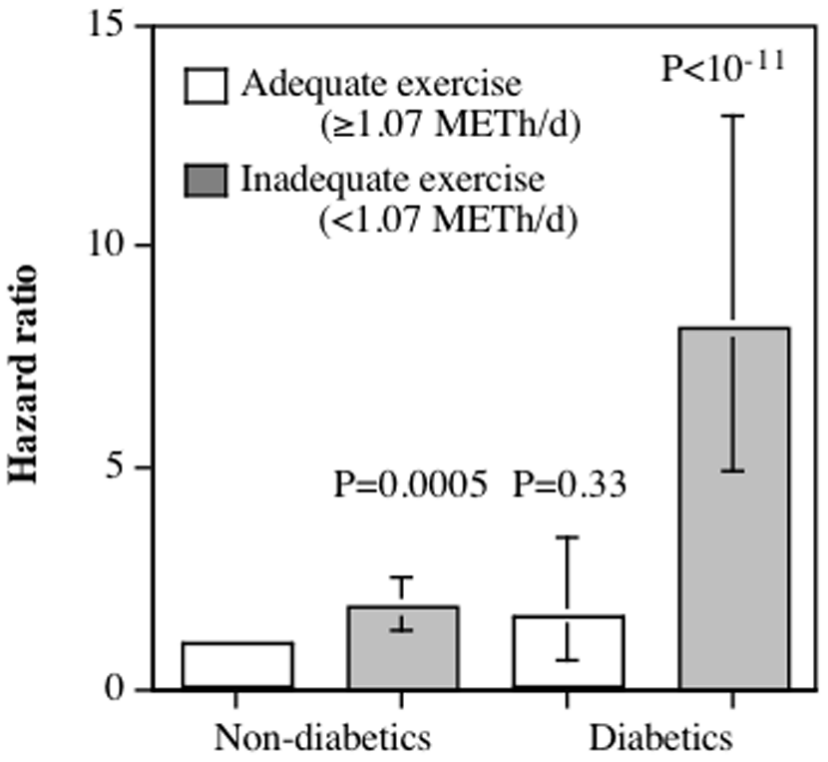
Relative risk of all sepsis-related mortality (sepsis_total_) in 133,324 non-diabetics who exercise adequately (≥1.07 METh/d), 20,000 non-diabetics who exercise inadequately (<1.07 METh/d), 1372 diabetics who exercise adequately, and 788 diabetics who exercise inadequately during an average of 11.6-year mortality surveillance. Brackets designate 95% confidence intervals. Relative risks (i.e., the hazard ratios) relative to non-diabetics who exercise adequately were calculated from Cox proportional hazard analyses adjusted for sex, age (age and age^2^), race, and cohort effects. Significance levels correspond to the significance of the risk increase relative to non-diabetics who exercise adequately.

There were 21 sepsis-related deaths in the 789 diabetics who did not exercise adequately (2.8%) and 6 sepsis-related deaths in the 1374 diabetics who exercise adequately (0.4%). Inadequate exercise was associated with greater sepsis_total_ mortality in the baseline diabetics (4.78-fold greater risk, 95%CI: 2.02- to 4.74-fold, P = 0.0002) when adjusted for age, sex, cohort, education, and race. Figure 2 shows that inadequate exercise was a significantly greater risk factor sepsis_total_ in diabetics than non-diabetics (P = 0.03). The proportion of total deaths that was sepsis-related was 13.0% the 162 inadequately exercising diabetics who died, which was significantly greater than the 3.6% the 169 diabetics who exercised adequately and died (2×2 Chi square, P = 0.002), showing that the increase in sepsis-related deaths with inadequate exercise was not simply due to differences in total mortality between exercising and non-exercising diabetics.

### Elders

Inadequate exercise in the 19,191 subjects who were 60 years of age or older at baseline was associated with a 1.98-fold increase in the risk of sepsis_underlying_ mortality, 1.72-fold increase in the risk of sepsis_contributing_ mortality, and a 1.79-fold increase in the risk of sepsis_total_ mortality (Table 2). In addition, those taking diabetic medication at baseline were at 2.98-fold greater risk of sepsis_total_ mortality (95%CI: 1.84- to 4.62-fold greater risk, P = 0.0001), whereas there was no significant effect of education (P = 0.40), years run or walked ≥19 km per week (P = 0.36), BMI (P = 0.53) or waist circumference (P = 0.18) on sepsis_total_ mortality in this older subset.

The effects of inadequate exercise on sepsis mortality was even more significant when the analyses were restricted to the 11,654 subjects who were 65 years or older at baseline, i.e., a 2.21-fold increase in the risk of sepsis_underlying_ mortality (95%CI: 1.07- to 4.54-fold, P = 0.03, 34 deaths), 1.88-fold increase in the risk of sepsis_contributing_ mortality (95%CI: 1.23- to 2.87-fold, P = 0.004, 96 deaths), and a 1.97-fold increase in the risk of sepsis_total_ mortality (95%CI: 1.36- to 2.83-fold, P = 0.0003, 130 deaths). For the 6406 subjects who were seventy or older at baseline, the corresponding increases in risk from inadequate exercise showed a 2.38-fold increase in the risk of sepsis_underlying_ mortality (95%CI: 1.07- to 5.41-fold, P = 0.03, 26 deaths), a 1.74-fold increase in the risk of sepsis_contributing_ mortality (95%CI: 1.10- to 2.74-fold, P = 0.02, 82 deaths), and a 1.88-fold increase in the risk of sepsis_total_ mortality (95%CI: 1.26- to 2.79-fold, P = 0.002, 108 deaths).

## Discussion

These analyses suggest that inadequate exercise is associated with a greater risk for sepsis mortality independent of other risk factors. The lack of prior studies showing a reduction in sepsis with physical activity and exercise may relate to reliance on exercise durations as a means for estimating energy expenditure. Specifically, there is a poor correspondence between self-reported time spent exercising and objectively measured exercise energy expenditure [30], [31], and we have shown that energy expenditure calculated from distance run or walked to be a superior metric to time base calculation [21]–[23]. The lower percentage of total deaths due to sepsis in our sample (4.8%) compared to 9% of all deaths in the United States [3] is likely due to a higher percentage of sepsis deaths in older people. Specifically, the average age of death in our sample was 70.2 for males (compared to 78.5 years old for 45 year old white males in the United States [32]) and 68.8 years in females (compared to 82.3 years old for 45-year old white females in the United States [32]).

### Mechanisms

Physically active individuals are known to be more responsive to vaccinations, have fewer exhausted or senescent T-cells, greater capacity for T-cell proliferation, lower circulating inflammatory cytokines, greater neutrophil phagocytic activity, less inflammatory response to bacterial challenge, greater NK-cell cytotoxic activity and longer leukocyte telomere lengths than those less active [13]. Collectively, these effects suggest that exercise is associated with less immunosenescence, *i.e.*, the decline in the normal functioning of the immune system with age [13]. These effects are less well-documented in training than cross-sectional studies [13], which might reflect the smaller exercise dose, shorter duration, or more limited statistical power achievable in training studies, or the tendency for those with greater immunity and inflammatory control to choose to exercise (i.e., self-selection). The harmful effects of severe sepsis are primarily due to the poorly moderated immunologic and coagulopathic response to an infection rather than the direct effects of the microorganisms themselves [33]. Exercise has been proposed as a model of temporary immunosuppression that occurs after severe physical stress [34], [35]. However, exercise does not elicit a fully-developed systemic pro-inflammatory response, which may be an adaptation to the cytokine response, an adaptation that may also minimize the systemic inflammatory response syndrome (SIRS) to sepsis [34], [35].

Suppression of immune function is thought to require exercise that is both prolonged and vigorous, e.g. for marathon participation but not shorter distance [34], [35], and is hypothesized to provide an opportunity for infection. Athletes report symptoms commonly associated with infections of the upper respiratory airways during heavy training and prolonged exercise [36]–[38], but it is possible that these are allergy- or inflammatory-based rather than infectious [39]. We found that sepsis mortality was unrelated to marathons run per year in 113,472 runners. In addition, we have previously reported that respiratory disease and pneumonia mortality decreased 7.9% and 13.1% per MET-hours/d run or walked, with no difference between running and walking [40].

### Effects of aging

Our analyses showed that most of the sepsis-associated mortality occurred in those sixty and older, consistent with age being a significant risk factor. Immunosenescence particularly affects adaptive immunity, i.e. T- and B-lymphocytes and their products. One hypothesis is that immunosenescence is in part a consequence of immune exhaustion from lifelong exposures to external pathogens and persistent viral infections [13]. It is not known whether exercise prevents immunosenescence or restores immunity lost as a consequence of immunosenescence [14]. However, the exercise-induced reduction sepsis mortality observed in this study was not significantly affected by how long the person had been exercising, suggesting that exercise did not mitigate exhaustion by lessoning lifetime exposure. Alternatively, exercise might prevent or revert the effects of age on the immune system. Leukocyte telomere length is considered an indicator of biological age [13], is associated with both morbidity and mortality [13], correlates positively with leisure-time physical activity [41], and shows differences between active and inactive subjects that correspond to about ten years of biological aging [35].

### Diabetes mellitus

Type 2 diabetes is associated with an increased susceptibility to infection and sepsis probably due to neutrophil defects that are likely due to hyperglycemia, and possibly defects in humoral immunity [42]. Our data shows that diabetes was an important risk factor for sepsis, and that inadequate exercise was associated with a nearly 5-fold increase in sepsis_total_ mortality in the total sample, and a 3-fold increase in risk for those 60 years and older. Others report that aerobic exercise training decreases plasma CRP, TNF-α and IL-18/IL-10 ratios in type 2 diabetics [43]. Figure 2 shows that the increased risk from inadequate exercise is significantly greater than in diabetics than non-diabetics, which could reflect in part the different effects of exercise on regulatory T-cells in type 2 diabetes mellitus and non-diabetic subjects [13]. Tregs, CD4+CD25+ cells that express Forkhead Box 3 (FoxP3), are lymphocyte-responsible for suppressing excessive immune responses. Exercise is reported to increase their FoxP3 expression in type 2 diabetes mellitus, but not healthy controls [44].

### Animal models

Intravenous lipopolysaccharide challenge and cecal ligation and puncture are common animal models for sepsis. Both have been used to show that exercise training mitigates sepsis response. Chen *et al.* showed that lipopolysaccharide-induced sepsis produced smaller increases in endotoxin-induced release of NO, free radicals, and pro-inflammatory cytokines (TNF-α and Il-1β) and less severe cardiac, hepatic, and pulmonary injury in exercise-trained than control rats [16]. Exercise-trained rats also more effectively counteract lipopolysaccharide-induced cardiovascular abnormalities and pulmonary edema than untrained controls [20]. Exercising rats to near exhaustion before intravenous lipopolysaccharide challenge also suppresses the systemic TNF response normally produced by the challenge [18]. Cecal ligation and perforation produced less atrophy, lipid peroxidation and protein oxidation and greater skeletal muscle superoxide dismutase activity in rats [17], and less lung and distal organ damage and greater survival in mice [19] following exercise training.

### Caveats and limitations

There are important limitations to these results. First, running, walking, and other baseline variables were self-reported from the participants' baseline questionnaires. In some cases, the reported exercise occurred a decade before the conclusion of the mortality surveillance. Exercise levels, and other subject characteristics could have changed prior to infection. There may also be residual confounding from socioeconomic status and other variables that were not entirely eliminated by the statistical adjustment for education and other variables in Table 2. Because the exercise performed was self-selected, it is not known whether exercise caused the reduction in sepsis death, or if persons with greater immunity and inflammatory control chose to exercise; however, various animal models support a causal effect [16], [17]–[20].

Our use of mortality is both a strength and weakness–: a strength in its ease of ascertainment, lack of subjectivity, and broad importance to patients and the critical care community, a weakness in that it is not known whether the risk reduction is due a lower risk of infection, a lower risk of mortality among those infected, or both. Another important limitation of these analyses is the lack of information on the circumstances of the infections. Death certificate diagnoses do not distinguish pneumonias acquired in the community, nursing homes, and health care facilities. Almost all of the diagnoses were pneumonia, unspecified ((ICD_9_486, ICD_10_J18.9). Differential mortality could represent differences in susceptibility to infection as well as the adequacy, timeliness, and response to antibiotic treatment [24]. Additional studies are required to track incidence, circumstances of infection, and treatment to determine whether the relationships observed are affected by patient physiology, behavior, or access to quality treatment in addition to exercise. Vital status is known only from the National Death Index and therefore some subjects who have died are likely to be misclassified as alive. Finally, we caution with regards to implying cause and effect in that individuals with greater susceptibility to respiratory disease may choose to exercise less.

Second, the dose-response relationship below 1.07 METh/d is not described. Included within the inadequate exercise group would be very sedentary individuals who may be responsible for the increased risk; however, the limited number of sepsis deaths to date precludes more detailed analyses within this group. Third, the specific pathogens giving rise to sepsis was not known for 93% of the sepsis_underlying_ causes of death, and 90% of the sepsis_contributing_ causes of death. It is also not known whether the sepsis was community-acquired, health care-associated, or nosocomial bloodstream infections. Fifth, we note that as only fatal sepsis was studied, these analyses cannot distinguish an etiologic from a prognostic effect of inadequate exercise. We also note that sepsis as an endpoint was not identified a priori and as a secondary hypothesis requires confirmation in additional studies. Finally, we caution that the current sample was recruited specifically to study the effects of walking and running on disease risk and was not selected to be representative of the general population. Additional studies in more representative epidemiological cohorts are required to establish the generalizability of exercise-sepsis relationship to the population at large.

### Conclusions

These results suggest that inadequate exercise is associated with a doubling of the risk for sepsis mortality. Given that the incidence of sepsis is increasing [3], these results suggest that getting people to exercise could be immediately effective in reducing the disease incidence.
